# Granulomatous Mastitis: A Clinical and Diagnostic Dilemma

**DOI:** 10.5146/tjpath.2021.01554

**Published:** 2022-01-21

**Authors:** Emel Ebru Pala, Sumeyye Ekmekci, Melis Kılıc, Ayberk Dursun, Gul Colakoglu, Cem Karaali, Mumin Emiroglu, Mustafa Emiroglu

**Affiliations:** Department of Pathology, Tepecik Education and Research Hospital, University of Health Sciences, Izmir, Turkey; Department of General Surgery, Tepecik Education and Research Hospital, University of Health Sciences, Izmir, Turkey; Department of Radiology, Tepecik Education and Research Hospital, University of Health Sciences, Izmir, Turkey

**Keywords:** Breast, Granulomatous mastitis, Idiopathic

## Abstract

*
Objective:* Granulomatous mastitis (GM) is a challenging inflammatory disorder of the breast. In this study we aimed to present the detailed clinical and morphological features of GM cases, diagnostic clues for specific and idiopathic etiologies, the difficulties in evaluating trucut biopsies, and the results of different therapeutic approaches.

*
Material and Method:* We retrospectively analysed the clinical, radiological and morphological features of 114 GM cases diagnosed with fine needle aspiration, and trucut, incisional, and excisional biopsy.

*
Results:* The mean age was 35.8. Only eight cases were older than 45 years. Bilateral involvement was observed in 4 (3.5%) cases. The most common clinical symptoms were breast mass/abscesses, tenderness, and skin changes. Microbiological culture was positive in 4 cases for gram-positive bacteria. Only 3 cases showed a positive tuberculin/PCR test for tuberculosis. The major USG finding was a hypoechoic well-defined or ill-defined mass/abscess; MRI finding was heterogeneous non-mass contrast enhancement. Cases diagnosed with cytology (35 cases) did not have breast malignancy either in their history or clinical follow up period. Fine needle aspiration cytology materials revealed epitheloid granulomas mixed with neutrophils, lymphocytes accompanied by giant cells, and suppurative necrosis. Histopathological reevaluation of 65 trucut/incisional/excisional biopsies revealed granuloma formation in 65 (100%), Langhans type giant cells in 59 (90.7%), microabscess formation in 41 (63%), caseous necrosis in 1 (1.5%), neutrophilic cysts in 30 (46.1%), eosinophilic infiltration in 48 (73.8%), interlobular inflammation in 14 (21.5%), fat necrosis in 5 (7.6%), ductal ectasia in 6 (9.2%), and lactational changes in 4 (6.1%) cases. Granulomas were lobulocentric in 58 cases, foreign body type/fat necrosis-related in 6 case, and periductular in 1 case. Cystic neutrophilic granulomatous mastitis was observed in one case. We also evaluated the histochemical stains of these 65 biopsies. Only one sample was positive for acido-resistant bacilli (ARB) by the EZN method and one sample was positive for gram-positive bacilli by gram stain.

*
Conclusion:* Small, superficial trucut biopsies may cause difficulties in determining the etiology and differential diagnosis of granulomatous mastitis. For optimal management and timing the appropriate therapy, the ideal biopsy procedure, special stains, and a multidisciplinary team consisting of the surgeon, pathologist, and radiologist are the most important issues.

## INTRODUCTION

Granulomatous mastitis (GM) was described as a benign inflammatory disorder in 1972 (1). It is characterized by lobulocentric destructive granulomatous inflammation relatively sparing interlobular stroma (2). GM may present with nipple discharge, orange peel sign and irregular masses mimicking breast carcinoma clinically and radiologically (2). Women with GM are usually parous and within 5 years of pregnancy (3). GM can be divided into two groups; idiopathic and specific. Gestation, oral contraceptive therapy, diabetes mellitus, smoking, autoimmunity are considered as the major etiological factors of idiopathic GM (4–6). Specific GM is due to the foreign body reaction, sarcoidosis, vasculitis, infectious causes such as tuberculosis, cat scratch disease, fungal inflammation, and corynebacteria infection (4). The definitive diagnosis can be made by histopathological examination.

The idiopathic form of GM is problematic for clinicians and surgeons in terms of the diagnosis and treatment strategy. The appropriate treatment modality depends on the etiology, extent of the lesion, and skin changes with fistula/abscess formation.

In this study we aimed to present the detailed clinical and morphological features, diagnostic clues for specific and idiopathic GM, difficulties in trucut biopsies, and the results of different therapeutic approaches.

## MATERIAL and METHOD

We retrospectively analysed the clinical, radiological and morphological features of 114 cases diagnosed as GM with fine needle aspiration (n=35) or trucut/incisional/excisional biopsy (n=79). None of the cases had breast malignancy in their history or during the clinical follow up period. The clinical data included age, clinical presentation, medical treatment history, and microbiological culture results. Radiological features were collected from the archived ultrasonographic, mamographic and MRI images. We obtained the hematoxylin & eosin stained slides and histochemical special stains of 65 cases. We reevaluated the histopathological features including the existence of granuloma formation, Langhans type giant cells, microabscess formation, caseous necrosis, neutrophilic cysts, eosinophilic infiltration, interlobular inflammation, fat necrosis, periductal inflammation, and ductal ectasia in 65 cases. Periodic acid schiff (PAS), Ehrlich-Ziehl-Neelsen (EZN), and Methanamine silver (MS) stained histochemical slides were also reviewed.

The study was approved by the Local Ethics Committee (2020/8-14).

## RESULTS

The mean age was 35.8 (min:19-max:76) years. Most of the patients were at the reproductive and childbearing age. Only eight cases were older than 45 years and three cases had a pregnancy/breastfeeding history.

Bilateral involvement was observed in 4 (3.5%) cases and the lesional breast was the left side in 63 (55.2%) cases. The most common clinical symptoms were breast mass/abscess (n=86), tenderness (n=34), skin changes (erythema, peau d’orange, ulcerated areas, sinus tracts) (n=15), and axillary mass (n=26) (Table 1). Microbiological culture was examined in 37 cases and 4 of them were positive for gram-positive bacteria. Only 3 cases showed a positive tuberculin/PCR test for tuberculosis.

**Table 1:** Clinical features of the cases.

**Clinical features**	**n (cases)**
Mean age	35.8 (min:19-max:76)
Bilaterality	4
Pregnancy /breastfeeding history	3
Breast mass/abscesses	86
Tender	34
Axillary mass	26
Skin changes	15

All patients were examined with ultrasonography (USG). USG revealed a hypoechoic well-defined or ill-defined mass/abscess (n=85), parenchymal heterogeneity/edema (n=12), parenchymal distortion (n=12), or dilated ductus (n=5). Mammography or MRI was added in 35 cases, because of suspected malignancy, bilaterality, or suspicious lymphadenopathy. The major MRI findings were non-mass forming heterogeneous contrast enhancement, microabscess, and edema.

Cases diagnosed with cytology (n=35) did not have breast malignancy either in their history or the clinical follow up period. All of them were diagnosed between 2000 and 2004, before the widespread use of trucut biopsy. Clinical and radiological features were concordant with granulomatous mastitis. Fine needle aspiration cytology materials revealed epitheloid granulomas mixed with neutrophils, lymphocytes accompanied by giant cells, and suppurative necrosis.

**Figure 1:**
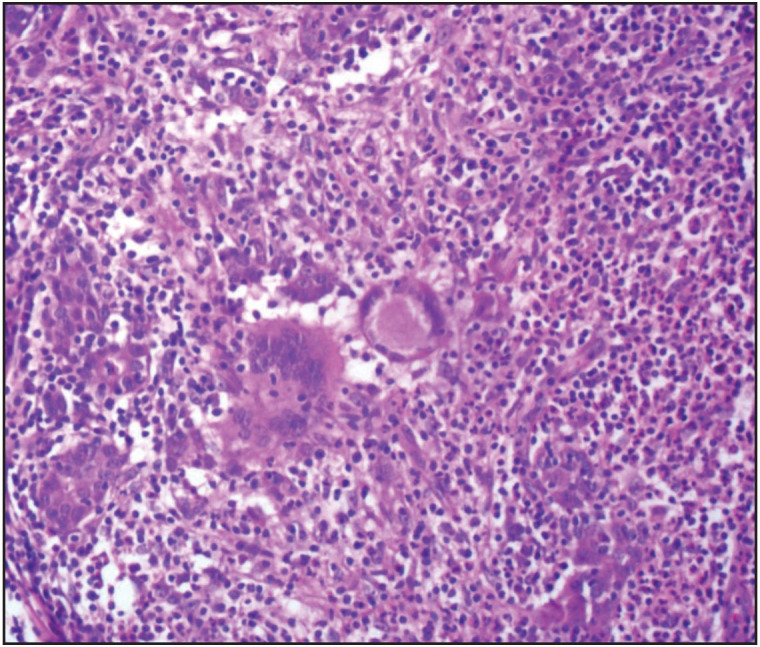
Lobulocentric granuloma formation with giant cells (HE, x200).

**Figure 2:**
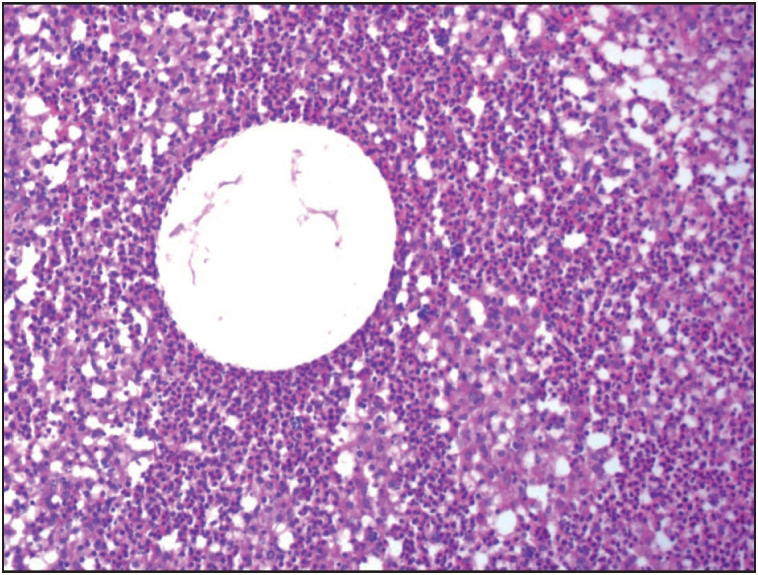
Central round cystic spaces rimmed by neutrophils and cuff of epithelioid histiocytes. (HE, x100).

**Figure 3:**
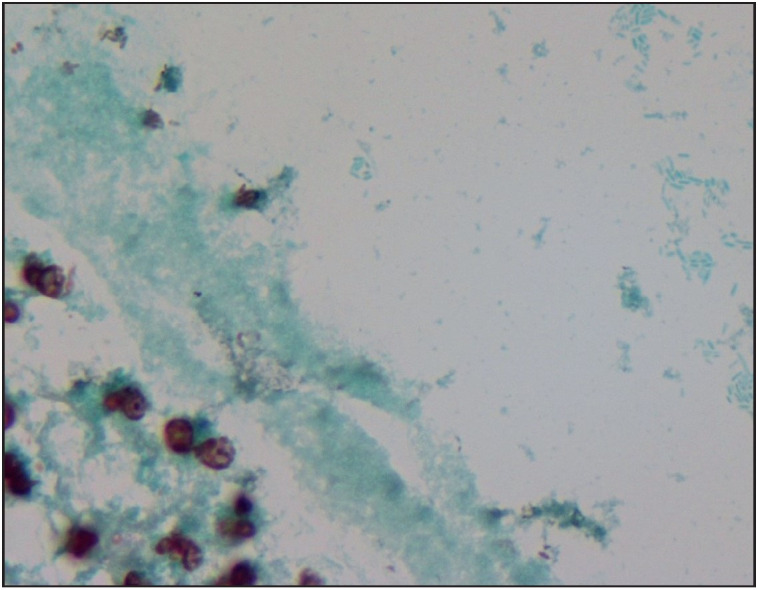
Some of the lipid vacuoles contain rod-shaped, gram-positive bacilli (Gram, x400).

Histopathological reevaluation of 65 cases revealed granuloma formation in 65 (100%), Langhans type giant cells in 59 (90.7%), microabscess formation in 41 (63%), caseous necrosis in 1 (1.5%), neutrophilic cysts in 30 (46.1%), eosinophilic infiltration in 48 (73.8%), interlobular inflammation in 14 (21.5%), fat necrosis in 5 (7.6%), ductal ectasia in 6 (9.2%), lactational changes in 4 (6.1%) (Table 2). Granulomas were lobulocentric in 58 cases (Figure 1), foreign body type/fat necrosis-related in 6 cases, and periductular in 1 case. Cystic neutrophilic granulomatous mastitis was observed in one case (Figure 2). We also evaluated the histochemical stains of these 65 cases for PAS, EZN and MS. One sample was positive for acido-resistant bacilli (ARB) with EZN, and one sample was positive for gram-positive bacilli with gram stain (Figure 3).

**Table 2:** Detailed histopathological features of 65 cases.

**Histopathological features**	**n (%)**
Granuloma formation · Lobulocentric · Foreign body/fat necrosis type · Periductular	65 (100) 58 (89.2) 6 (9.2) 1 (1.6)
Langhans type giant cells	59 (90.7)
Microabscess formation	41 (63)
Caseous necrosis	1 (1.5)
Neutrophilic cysts	30 (46.1)
Eosinophilic infiltration	48 (73.8)
Interlobular inflammation	14 (21.5)
Fat necrosis	5 (7.6)
Ductal ectasia	6 (9.2)
Lactational changes	4 (6.1)

Treatment options were medical therapy (steroid, antibiotics, antituberculous therapy), abscess drainage, and excision. Medical therapy was the first choice in large, complicated lesions; and surgery in localized lesions. Patients were given oral antibiotics during the biopsy procedure, and then started steroid therapy. Steroid therapy was gradually reduced and then discontinued. None of the cases were treated with immunosuppressives. The cases with ulceration, fistula formation, and affecting the skin area had undergone excision.

Initial treatment option was medical therapy in 65, abscess drainage+medical therapy in 15, affected skin excision+medical therapy in 4, and lumpectomy in 30 cases. Recurrence was observed in 15 (13.1%) cases, and 10 of them were from the medical therapy group. Recurrent excisions were performed in 6 cases.

## DISCUSSION

The etiology of GM is elusive in most cases and it presents as a diagnostic and therapeutic dilemma. Mycobacterial, fungal, parasitic disease, sarcoidosis, IgG4-related sclerosing disease, and autoimmune diseases can involve the breast and show a granulomatous inflammation pattern (7). Also foreign body reaction, fat necrosis, duct ectasia, epidermal cysts of the nipple skin, squamous metaplasia of lactiferous ducts (SMOLD), and cystic neutrophilic granulomatous mastitis should be considered in the differential diagnosis and ruled out with clinical, radiological and pathological features (7). In granulomatous lobular mastitis (GLM), granulomas centered in the lobules are deeply located in breast parenchyma. Fine needle aspiration biopsies (FNAB) and small, superficial trucut biopsies may lead to difficulties in identifying lobulocentric granulomas, the etiology of the granulomas, and the differential diagnosis. FNAB is a quick, noninvasive method with low sensitivity (8). It may be useful for excluding malignancy and obtaining a sample for microbiological analysis but is usually ineffective to find out the underlying cause. Core needle biopsies are more useful for a definitive diagnosis (9). However, granulomas may not be identified on core biopsy in up to 15% of the cases (4), especially the ones with intense microabscess formation masking the granuloma. Incisional and excisional biopsies are helpful to identify the localization of the granuloma, and for architectural evaluation.

In the differential diagnosis, the clinical features are also helpful. SMOLD is associated with smoking although GLM is not (10). Unlike GLM, duct ectasia and related granulomas occur in older woman (11). Etiopathogenesis of duct ectasia is obscure, but weakened duct walls is probably the primary process leading to rupture and release of duct secretions into the stroma. Periductal chronic inflammatory reaction and foreign body reaction occurs against the secretions. Ruptured epidermal cysts in the superficial dermis of the nipple skin may also cause foreign body type reaction and granulomas. In cases of extensive foreign body giant cell reaction, the classic features may not be visualized. Keratin flakes may lead to the diagnosis in superficial biopsies. In fat necrosis, the clinical history, lipid-laden foamy histiocytes, and multinucleated foreign body type giant cells are diagnostic. Our cohort also included granulomas due to ruptured epidermal cyst in one case, ductal ectasia in one case, and fat necrosis in five cases. However, the etiology of granulomas was overlooked in these seven cases at primary evaluation.

**Figure 4:**
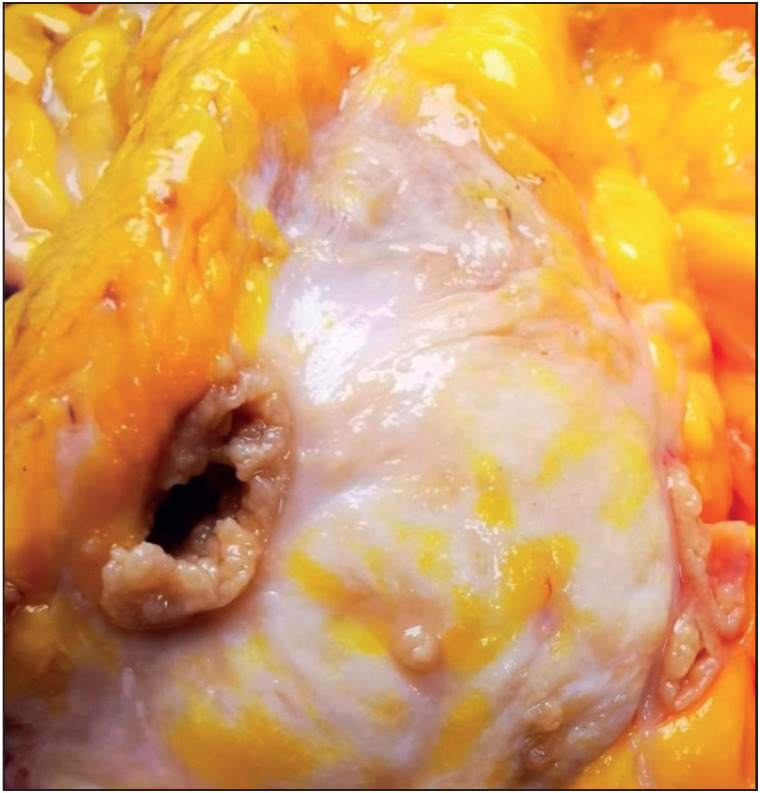
Excision material of cystic neutrophilic granulomatous mastitis case due to fistula and sinus formation.

Cystic neutrophilic granulomatous mastitis (CNGM) is characterized by lobulocentric mixed inflammatory infiltrate composed of lymphocytes, neutrophils and multinucleated giant cells; central round cystic spaces (lipid vacuoles) rimmed by neutrophils and a cuff of epithelioid histiocytes. Some of the lipid vacuoles may contain rod-shaped, gram-positive Corynebacterium bacilli (12). One should be aware of the difficulties in detecting corynebacterium by Gram stain or microbiological culture. Targeted microbiological techniques may be necessary for the detection (13). Central lipid spaces surrounded by neutrophils are not specific for CNGM, and should be investigated for fungal, mycobacterial and other bacterial organisms (13). We observed these vacuoles in nearly half of the cases and especially those including granulomas with microabscess formation. We concluded that cystic lipid vacuoles are also common in idiopathic granulomatous mastitis (IGM). The choice of antibiotic therapy and the optimal treatment duration still require further investigation. Only one CNGM case existed in our cohort, and showed resistance to prolonged antibiotic and steroid treatment. The case underwent surgical excision due to fistula and sinus formation (Figure 4).

Tuberculosis mastitis affects ducts rather than lobules and is composed of necrotizing or nonnecrotizing granulomas. EZN staining, culture or polymerase chain reaction can be used for establishing the diagnosis (11,14). In our cohort, three cases were positive for tuberculosis serologically, but only one of them showed caseous necrosis and acido-resistant bacilli with EZN. The granulomas were associated with both lobules and ducts. Lacambra et al. reported that tuberculosis mastitis tends to show more eosinophils and necrosis, but IGM is associated with more plasma cells (11). We did not notice such a difference in our study group. The features of the granulomas and giant cells were not useful for distinguishing IGM and tuberculosis. Cases were given anti-tuberculosis therapy for six months and there has been no recurrence during follow-up period.

**Figure 5:**
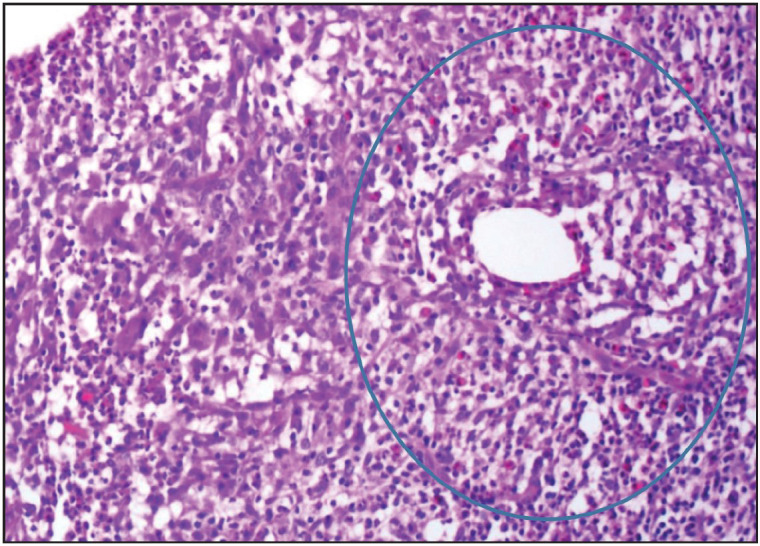
Eosinophils in mixed inflammatory component of granulomatous inflammation (HE, x200).

Eosinophils are rare in the mammary gland in breast carcinoma as well. They are more commonly seen in inflammatory conditions of the breast (15). Eosinophilic mastitis is an extremely rare condition characterized by heavy eosinophilic infiltrates around the ducts and lobules. Few cases of eosinophilic mastitis have been reported in the literature. Most of them were accompanied with hypereosinophilic syndromes (16). The pathogenesis is unknown, but localized allergic reaction to intraluminal substances is accused (16). We noticed significant numbers of eosinophils in the mixed inflammatory component of granulomatous inflammation in the majority of GM cases (Figure 5). This infiltration may be related to the efflux of protein secretions into the intralobular stroma.

When the patients had complaints of inflammatory breast disease, short-term antibiotic therapy was started. At the same time, baseline imaging was performed. Due to conflicting clinical and radiological features, core biopsy confirmation was preferred. If the biopsy findings revealed granulomatous mastitis, specific etiological factors were investigated. Oral corticostreoid therapy was started if all specific causes were excluded. The optimal treatment strategy is controversial in IGM. Nearly half of the cases develop a chronic course. Surgical complications may lead to lesions that are worse than the primary lesion (17). Recent approaches are biopsy, abscesses drainage, and complex fistula excision rather than complete excision. However, surgery is still also acceptable in selected cases.

Nearly half of the cases with small lesions and mild clinical symptoms resolve spontaneously within 1-2 years (18). This group may be suitable for close observation. Surgical excision should be performed in localized and uncomplicated lesions with clear margins (19). Cases with large, diffuse, complicated lesions are not suitable for surgery. Medical therapy should be preferred in large, diffuse lesions to reduce the size of the lesion and prepare the case for surgery. Steroids should be the first choice in complicated and recurrent cases. Methotrexate is appropriate for cases resistant to maximal dose of corticosteroids.

Lei et al. have reported low recurrence rates (4%–6.8%) with surgical managements with or without oral steroids (20). Recent studies recommend a conservative approach. Shin et al. reported higher recurrence rates (25%) in the excision group than the steroid and drainage group (7.1%). Additional steroid therapy seems like a better choice in recurrent cases because wider excisions lead to scarring with deformation (21).

Close clinical observation was not the choice in the cohort. Medical therapy was the first choice in 65 cases of which 10 showed recurrence. Recurrence after medical therapy was higher than with surgery. Affected skin excision/drainage combined with medical therapy was performed in 19 cases and seemed to be an effective modality.

The first step of the diagnostic algorithm is excluding the breast malignancy. Trucut biopsy should be preferred as the first choice but open biopsies may be required. The ideal biopsy procedure, special stains for the diagnosis, and the etiology of the granulomas are the most important issues for optimal management and timing the appropriate therapy.

## Conflict of Interest

The authors have no financial or personal relationships with other people or organizations to disclose that could have appeared to influence the work reported in this paper.
